# Reliability of the Resonance Frequency Analysis Values in New Prototype Transepithelial Abutments: A Prospective Clinical Study

**DOI:** 10.3390/ijerph17186733

**Published:** 2020-09-16

**Authors:** María Guerrero-González, Francesca Monticelli, David Saura García-Martín, Mariano Herrero-Climent, Blanca Ríos-Carrasco, José-Vicente Ríos-Santos, Ana Fernández-Palacín

**Affiliations:** 1 Periodontics, Faculty of Health and Sport Sciences, Universidad de Zaragoza, C/Velódromo S/N, 22006 Huesca, Spain; drmguerrero@gmail.com (M.G.-G.); fmontice@unizar.es (F.M.); clinicasaura@gmail.com (D.S.G.-M.); 2 Porto Dental Institute, 4150-518 Porto, Portugal; dr.herrero@herrerocliment.com; 3 Advanced Periodontics, School of Dentistry, Universidad de Sevilla, C/Avicena S/N, 41009 Sevilla, Spain; brios@us.es; 4 Department of Social and Health Sciences, Universidad de Sevilla, 41009 Sevilla, Spain; afp@us.es

**Keywords:** implant stability, osseointegration, implantology, implant design

## Abstract

Resonance frequency analysis (RFA) requires abutment disconnection to monitor implant stability. To overcome this limitation, an experimental transepithelial abutment was designed to allow a SmartPeg to be screwed onto it, in order to determine the prototype abutments repeatability and reproducibility using Osstell ISQ and to assess whether implant length and diameter have an influence on the reliability of these measurements. RFA was conducted with a SmartPeg screwed directly into the implant and onto experimental abutments of different heights of 2, 3.5 and 5 mm. A total of 32 patients (116 implants) were tested. RFA measurements were taken twice for each group from mesial, distal, buccal and palatal/lingual surfaces. Mean values and SD were calculated and Intraclass Correlation Coefficients (ICC) (*p* < 0.05, IC 95%). The implant stability quotient (ISQ) mean values were 72.581 measured directly to implant and 72.899 (2 mm), 72.391 (3.5 mm) and 71.458 (5 mm) measured from the prototypes. ICC between measurements made directly to implant and through 2-, 3.5- and 5-mm abutments were 0.908, 0.919 and 0.939, respectively. RFA values registered through the experimental transepithelial abutments achieved a high reliability. Neither the implant length nor the diameter had any influence on the measurements’ reliability.

## 1. Introduction

Implant stability is an essential requirement to achieve osseointegration and to ensure implant success over time [1]. Several non-invasive tests have been described to assess this stability [2]. Resonance frequency analysis (RFA) is an easy, non-invasive and reproducible method commonly used for this purpose. It is considered an implant-bone complex bending test, where a transducer applies an extremely small lateral force, simulating a clinical condition of load to a very small magnitude [3,4,5]. RFA is performed through Osstell system device (OSSTELL AB, Göteborg, Sweden) and converted into an Implant Stability Quotient (ISQ) value. This device is considered a high reliable and objective tool for measuring implant stability at the time of placement as well as during treatment and follow-up [6,7,8].

Current Osstell systems require the use of a SmartPeg, which is a magnetized transducer (SmartPeg OSSTELL AB, Göteborg, Sweden) that is screwed onto the implant and is designed specifically for each type of implant. This SmartPeg is capable of being stimulated by a magnetic impulse from a probe. After being excited, it vibrates and emits an electric voltage to the probe that will be transformed into an ISQ value. ISQ values seems to be influenced by several factors such as bone implant contact (BIC), which in turn is influenced by implant morphology and surface properties, among others; implant design, length, and diameter [4]; force at which the transducers are screwed (4–5 N/cm are recommended) [9,10,11]; interposed soft tissue between the SmartPeg and the implant [4]; bone quality [12]; probe position and effective implant length (EIL), which is defined as the distance from the transducer to the marginal bone (the greater the distance, the lower ISQ values are obtained) [4,10,13,14].

Current implantology is aimed at reducing clinical procedures, trying to establish individualized protocols without thereby jeopardizing implant viability. Immediate implant loading has become a common clinical procedure whenever a series of guidelines such as implant insertion, torque greater than 30 N and ISQ values above 60 among others are met. However, in the absence of these clinical requirements, other options, such as early loading (between 1 week and two months) has to be preferred [15]. In the same way, non-submerged technique using transepithelial abutments instead of submerged protocol is preferred if clinical conditions are achieved [16,17]. It seems to have been demonstrated that during the early healing process there is a decrease in implant stability, which corresponds to the decrease in primary stability and the gradual increase in secondary stability. This decrease in total stability corresponds to 2–5 weeks after installation, depending on factors such as implant surface or bone quality among others [17]. On one hand, during this period of low stability, the application of reverse torsional forces around 10 Ncm for the connection and disconnection of the abutments to perform RFA and to find out if the implant ISQ values are compatible with the prosthetic load (above 60 ISQ) can affect the healing process [18,19]. On the other hand, recent studies highlight that implant transepithelial abutments disconnection and reconnection can compromise and damage implant surrounding soft tissue leading to connective tissue and underlying bone loss as a consequence of injuries at the mucosal barrier level [20,21,22].

An experimental healing abutment prototype has been designed (SOADCO SL. Andorra) to allow a SmartPeg number 49 (OSSTELL AB, Göteborg, Sweden) to be screwed directly on it. These new abutments may optimize the procedure required to assess implant stability and proper loading time, reducing the anti-clockwise forces applied to the implant during the early healing period. This is in addition to reducing the number of abutment disconnections and reconnections to check out if the stability values are correct to proceed with the loading during the implant healing period. On the other hand, they may simplify RFA registration since the transducer is screwed more coronally, thus decreasing the possibility of soft tissue trapping. However, the registration of ISQ values may be compromised as a consequence of the increased distance between the SmartPeg and the bone crest (EIL) (if compared with the conventional RFA measuring procedure) improving micro-motion during measurement and therefore resulting in lower ISQ values [4]. Nonetheless, recent in vitro studies assessed the experimental abutments reliability with ISQ values similar to those obtained when RFA is performed directly to the implant. Therefore, the clinical assessment of the reliability of the experimental abutments would be a matter of interest [23].

The aim of this study was to determine the clinical reliability of prototype transepithelial abutments using the Osstell ISQ device by evaluating whether the ISQ values achieved are comparable to those obtained when the SmartPeg is screwed directly to the implant and to assess whether implant length and diameter have influence on the reliability of these measurements.

## 2. Materials and Methods

A cross sectional prospective in vivo clinical study was designed to evaluate repeatability and reproducibility of Osstell ISQ device measurements taken directly to the implant or through an experimental transepithelial abutment assessing whether these measurements are comparable.

The present study was carried out among patients attending the Dental Clinic of the University of Zaragoza (Spain) that were planned to receive an implant-supported prosthesis in the master’s degree in Periodontics and Oral Implantology. The clinical investigation protocol was approved by the ethical committee (approval number PI16/083—Committee of Research Ethics of the Autonomous Community of Aragon (CEICA), Spain). All procedures performed in studies involving human participants were in accordance with the ethical standards of the institutional and/or national research committee and with the 1964 Helsinki declaration and its later amendments or comparable ethical standards. Informed consent was obtained by all patients. Patients were recruited at three different times of their treatment: immediately after implant surgery, during prostheses manufacturing or in any maintenance visit. All the measurements were taken between June and November 2016. The stage in which the assessment was accomplished was not considered as relevant, since the purpose of this study was to evaluate the correlation between RFA measurements without taking into account the time of measurements.

The following inclusion criteria had to be met:

- Patients with age equal to or greater than 18 years.
- Patients whose wishes and needs were rehabilitation with dental implants.
- Collaborative patients with unremarkable medical histories (ASA 1)
- Healthy, non-smoking, no or minimal alcohol use [24] and not known diagnosed allergies.
- Implants that were placed in areas in which extractions were performed at least 4 months before the implant surgery, thus the ridge was fully healed.
- Implants placed in areas with no bone regeneration needed.
- Implants with absence of clinical mobility or painful symptoms.

Sample size was calculated trough n Query Advisor 4.0. (Statistical Solutions Ltd., cork, Ireland); the variance of the means was calculated, and the highest standard deviation (3.94) was used to ensure that all groups were covered. A significance level of 0.05 was established, using 4 groups (direct to implant, 2, 3.5, and 5 mm), with a variance between the means of 1.529 and a standard deviation of 3.94, assuming a power of 80%. The result obtained was n = 29 implants per group, as there were 4 groups (n = 116).

A total of 116 screw-shaped implants (Essential Cone, Klockner implant system, SOADCO S.L, Andorra) with rough surface (Shot Blasting: alumina particle sandblasting and acid passivation), placed in 32 patients in all locations of the oral cavity, anterior and posterior maxilla and mandible, were consecutively admitted in the study. Implant diameter was either 3.5, 4.0, 4.5 or 4.8 mm with a 4.5 mm platform and 1.5 mm mechanical neck. The implant length was either 8, 10 or 12 mm.

**Figure 1:**
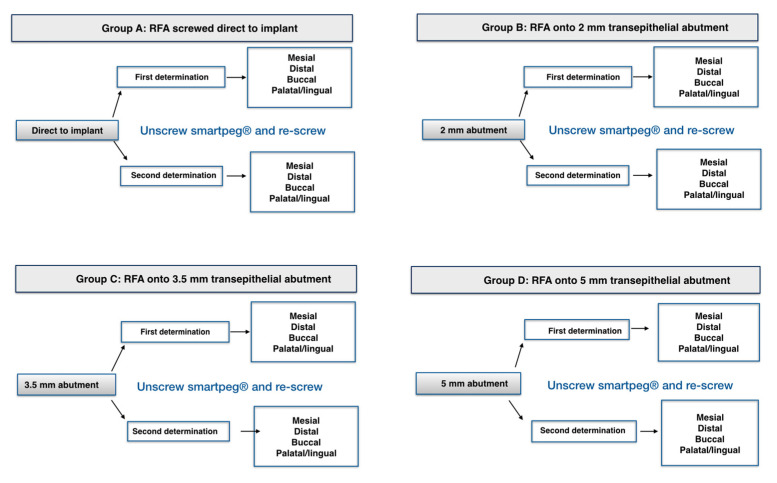
Resonance frequency analysis (RFA) registration protocol.

Implant stability in terms of RFA was assessed with the Osstell ISQ System and was measured in four different situations. In the first group (Group A), the stability was measured with the SmartPeg screwed directly to the implant. Then, the implant stability was registered screwing the SmartPeg on the top of three different height healing abutments (Group B: 2 mm, Group C: 3.5 mm and Group D: 5 mm). For each implant, four records were taken with the probe (mesial, distal, buccal and palatal/lingual), maintaining it with an angle of 90° with the implant major axis and at a distance of approximately 2 mm from the SmartPeg. In each situation, measurements were taken twice, unscrewing and re-screwing the SmartPeg in between, thus yielding a total of 32 measurements for each implant (Figure 1). All the measurements were made by the same researcher who had more than 5 years of experience in the use of the device. The examiner was calibrated one month before the beginning of the study by taking records on 10 implants, repeating these measurements in a different random order. A Kappa analysis was performed, and intraclass correlation coefficient was 0.915.

- A prototype transepithelial abutment was screwed at 10 N to avoid loosening by mechanical torque control as recommended by the manufacturer.
- Smartpeg placement was carried out strictly following manufacturer’s guidelines.
- No tissue interposition between implant and SmartPeg.
- Digital tightening (4–5 N/cm) with the plastic screwdriver provided by the manufacturer [25].
- The SmartPeg had no contact with the neighboring teeth.

**Figure 2:**
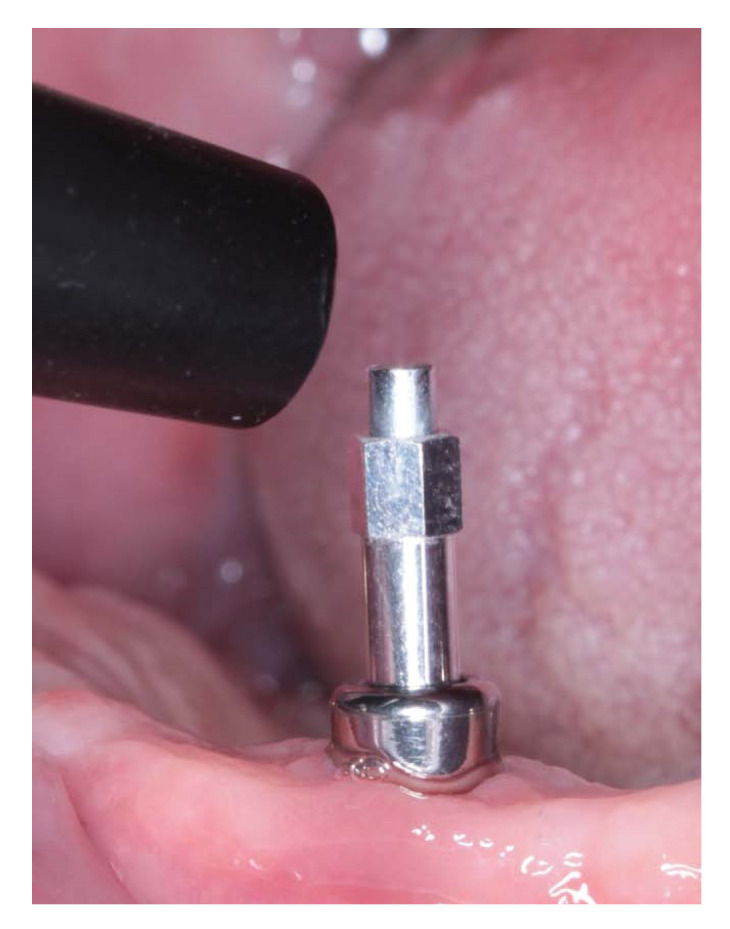
Transepithelial abutment and SmartPeg screwed onto de implant.

The experimental transepithelial abutments (SOADCO, S.L, Les Escaldes–Engordany, Andorra) were designed specifically for this study allowing to be screwed directly to the implant as well as providing a specific SmartPeg to be screwed in their inner part to perform RFA measurements with Osstell ISQ System device trough SmartPeg number 49, (OSSTELL AB, Göteborg, Sweden) (Figure 2).

**Figure 3:**
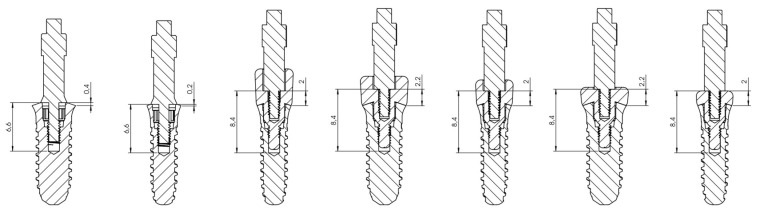
SmartPeg position is always constant, which translates into 1.8 mm with respect to the position it would have if the SmartPeg was screwed directly to the implant. Due to the distance between the implant platform and the lower part of the implant internal thread. Considering this as a fixed point, this distance is 6.6 mm when the SmartPeg is screwed directly to the implant; however, when we use the prototype transepithelial abutments, this distance is increased to 8.4 mm, being the same in all the transepithelial abutments resulting in an increase of the effective implant length (EIL) of 1.8 mm.

**Figure 4:**
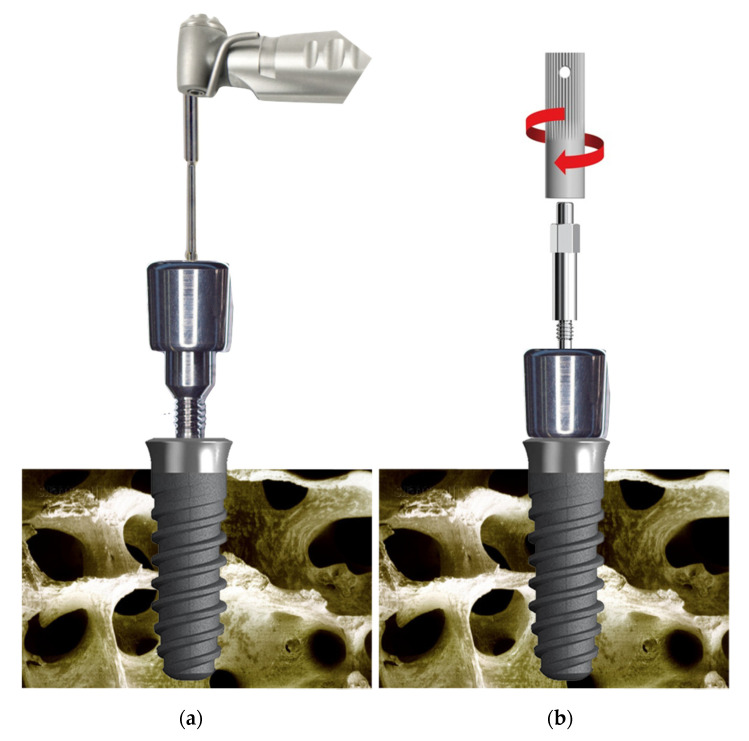
(**a**) Image of the healing cap placement method. (**b**) Manual placement of the SmartPeg with its plastic carrier.

**Figure 5:**
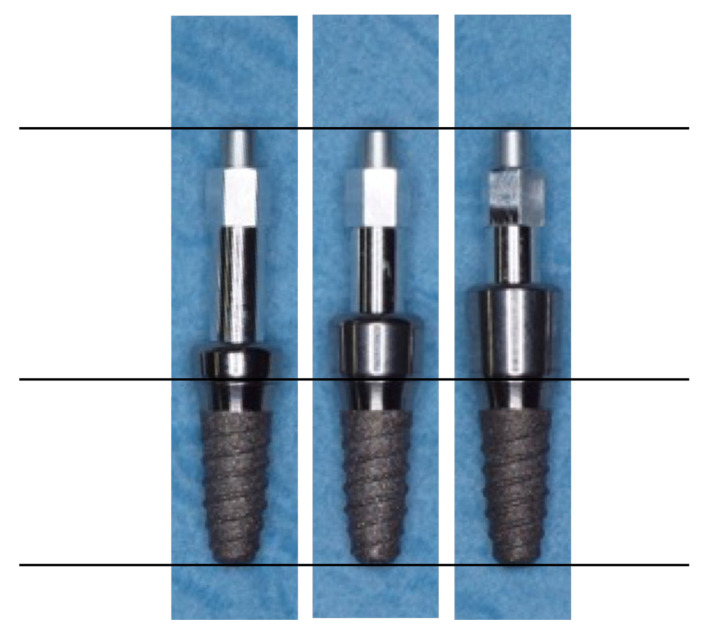
Healing plugs of different heights (2/3.5/5 mm) placed on the implant; it can be seen that the total height of the SmartPeg is the same (distance to the bone/implant shoulder) so as not to distort the resonance frequency analysis (RFA).

The abutments were manufactured in three different heights (2, 3.5 and 5 mm) and 4.5 mm wide specifically for Kolckner Essential Cone. The prototype transepithelial abutments have 3 parts: the external thread to be screwed to the implant, which has the same metric of a regular transepithelial 0.35 mm, the intermediate portion where the SmartPeg is screwed and has a height of 1.8 mm and a metric thread of 0.35 mm, and the upper portion where the SmartPeg is seated. Prototype abutments are made of Grade 5 titanium according to ASTM F136 (titanium-6aluminum-4vanadium). They have been machined on a numerical control lathe (CNC), and they are manufactured in the same way as the standard transepithelial abutments, having the same internal and external thread metric. Thanks to their design, the SmartPeg may be screwed as deep as possible inside the healing abutment, which allows it to be positioned as close to the bone as possible. Independently from the height of the healing abutments, the SmartPeg is located 1.8 mm farther from the bone than when it is screwed directly to the implant (Figure 3, Figure 4 and Figure 5).

Statistical analyses were performed using IBM Corp. Released 2016. IBM SPSS Statistics for Windows, Version 24.0. Armonk, NY: IBM Corp. Mean values and standard deviations were calculated. To study consistency among the different consecutive measurements provided by the same instrument on the same patients, the intraclass correlation coefficients (ICC) were calculated according to the model of analysis of variance with repeated or intra-subject measurements. Together with ICC´s, their intervals at 95% confidence (*p* < 0.05) were determined. ICC has been accepted as the concordance index for continuous data and the values obtained with the ICC range between 0 (absence of agreement) and 1 (absolute agreement), there being a certain consensus when accepting the following criterion: 0.01–0.20 Slight; 0.21–0.40 Average; 0.41–0.60 Moderate; 0.61–0.80 Substantial; 0.81–1.0 Almost Perfect. [6,26]

The same protocol was used to analyze the reliability of the RFA determinations using ICC to evaluate measurements repeatability/reproducibility according to implant height, implant diameter and the influence of height and diameter taking them simultaneously.

### Ethical Approval

All procedures performed in studies involving human participants were in accordance with the ethical standards of the institutional and/or national research committee and with the 1964 Helsinki declaration and its later amendments or comparable ethical standards. The clinical investigation protocol was approved by the ethical committee (approval number PI16/083) (Committee of Research Ethics of the Autonomous Community of Aragon: CEICA-Spain-). Informed consent was obtained by all patients.

## 3. Results

Measurements were carried out in 116 implants (32 patients). All implants were clinically stable, no symptoms and mobility were recorded. 40% of implants were located in posterior maxilla, 20% in anterior maxilla, 28% in posterior mandible and 12% in anterior mandible. Implant length was distributed as follows: 9, 66 and 41 implants of 8, 10 and 12 mm, respectively, and implant width distribution was 48, 52 and 16 implants of 3.5, 4 and 4.5 mm, respectively.

Mean ISQ values according to where the SmartPeg was screwed were 72.58 when the SmartPeg was screwed directly to the implant; 72.89, 72.39 and 71.46 when the transducer was screwed on 2-, 3.5- and 5-mm height abutments, respectively. RFA measurements expressed in ISQ units and registered screwing the SmartPeg to 2-, 3.5- and 5-mm height abutments had an almost perfect correlation when compared with the RFA measurements carried out with the SmartPeg screwed directly to the implant, with an ICC of 0.90, 0.92 and 0.94, respectively. The measurements achieved when the transducer was screwed to the experimental abutments (2, 3.5 and 5 mm) have an almost perfect correlation when ICC is applied (0.99, 0.97, 0.98, respectively). The RFA records obtained in all groups revealed a very good correlation and an almost perfect repeatability and reproducibility.

RFA mean values expressed in ISQ units, achieved twice and from each side (mesial, distal, buccal and palatal/lingual) using the SmartPeg screwed directly to the implant and to the prototype healing abutments, are summarized in (Table 1).

In all groups, RFA measurements carried out twice from mesial, distal, buccal and palatal/lingual sides showed an almost perfect reliability with ICC values, showing very high correlations, with very homogeneous intervals (Table 2).

Implant length (8, 10 or 12 mm) (Table 3) or implant diameter (3.5, 4 or 4.5 mm) (Table 4) had no influence on the reliability of the RFA records, independently from the measuring technique (directly to the implant or through 2-, 3.5- and 5-mm transepithelial abutments) nor when compared the records obtained with the different transepithelial abutments among themselves. Data also revealed that implant length and diameter evaluated simultaneously have no influence on repeatability and reproducibility of RFA records, independently from the measuring procedure. Eight-mm implants were excluded for this determination due to the reduced sample size.

Since there was an almost perfect correlation between the first and the second measurement in all the experimental groups, the mean ISQ values were studied (first and repeated measurement), and ICC were carried out to assess the different groups correlation registered for the different probe positions. Results revealed an almost perfect correlation (Table 5).

## 4. Discussion

Different studies reported the usefulness of the RFA Osstell system to achieve an objective evaluation of implant stability, as a consequence of the high reliability of its results and its greater reproducibility and repeatability compared to other methods developed with a similar purpose [4,6,27,28,29]. The results obtained in this study showed that ISQ values recorded with the healing abutment (2, 3.5 and 5 mm) have a very good reliability (ICC = 0.91/0.91/0.93) compared to values registered directly to implant. These results are in agreement with those obtained by Herrero-Climent et al. that assessed the reliability of the measurements with Osstell ISQ on 85 implants, yielding an ICC of 0.97 [7]. Moreover, records taken using the transepithelial abutments of different heights reported a very good correlation between them.

The experimental healing abutment could be used for the same indications as regular abutments; they would be indicated in those situations in which one stage surgery protocol can be performed. The two-stage technique is associated with the need to perform regenerative techniques, the use or removable provisional prostheses (to prevent excessive load transmission to the abutments) and when implant primary stability levels are not high enough. In this regard, Baltayan et al. demonstrated the importance of the use of RFA at the time of implant placement in the decision-making of a submerged or non-submerged protocol, stating that ISQ of 66 could serve as a “cut-off” value in the decision making of the transepithelial abutment placement the day of surgery [17], so the use of the experimental healing abutments would be indicated in those cases in which the ISQ values are higher than 66 on the day of surgery.

Although RFA measurements through transepithelial abutments have been implemented recently, the technique has not been validated so far. Our results differ substantially from those obtained by Lages et al. where in an in vivo study over 31 implants investigated the relationship between the ISQ values with different prosthetic abutments and with the implant platform. They conclude that the higher the abutment, the lower ISQ value [30]; this may be due to the statistical test and the different abutment design used for their study.

ICC has been accepted as the continuous data concordance index. If the size of the components of the variance between and within the groups is evaluated, the ICC describes the proportion of the total variation that is explained by the differences between the observers and measurement instruments [31]. Random effects patients and a fixed observer represent a mixed model problem, and therefore, according to the classification of Shrout-Fleiss, it is an ICC. The mathematical definition of the ICC is derived from a variance model of mixed analysis. The total variance between measurements is due to three different sources: the difference between observers, the differences between subjects under study and the residues that represent the unexplained variation [26].

The term effective implant length (EIL) was first introduced by Meredith et al. in 1996, assessing that there was a strong correlation (r = 0.94, *p* < 0.01) between resonance frequency values and the implant length above the bone crest. Therefore, they concluded that RFA could be used for peri-implant bone loss monitoring [3]. Tözum et al. also concluded that the Ossell System is able of discriminating bone losses as small as 1 mm, which means that a minimum EIL increment can produce significant decreases in resonance frequency values [32]. This is in disagreement with the results obtained in this investigation where an EIL up to 1.8 mm for Klockner Essential Cone implant did not influence the ISQ values achieved, although it is necessary to point out that Tözum´s et al. studied was carried out in “in vitro” conditions on resin models, and our work was in vivo. However, our results are in accordance with Merheb et al. and Lachmann et al. Both studies were performed in vitro on acrylic models and evaluated the Ostell System capacity to detect marginal bone loss. They both concluded that a minimum loss of 2 mm is necessary to be detected. This translates into an increase in the EIL of 2mm without altering the ISQ values [33,34]. The relationship between implant stability measured by RFA and bone loss during early healing period was studied by Yang SM et al. in vivo. They concluded that radiographic losses of 1.28 +/− 0.51 mm and 1.32 +/− 0.57 mm on the mesial and distal sides did not change the mean implant stability quotient values [35]. More studies are needed to clarify the influence of EIL on ISQ values in vivo.

The results of this study reveal that the probe orientation (mesial, distal, buccal, palatal/lingual) has no influence on ISQ values when records are measured with the SmartPeg screwed directly to the implant or to the experimental transepithelial abutments. These findings are in consonance with those published by Sim and Lang [14] although differ from previously published results that considered the probe orientation is able to influence resonance frequency values [10,34,36]. Although there is no statistically significant difference between the probe position (*p* < 0.5), a decrease in ISQ values may be registered in the vestibular position for all the tested groups: This may depend on the fact that buccal plates usually are thinner [37]. No registrations were made from the occlusal aspect since it seems to induce a decrease in resonance frequency values of 8 to 10 points with respect to other orientations. As a consequence, the manufacturer tends to discourage the registration from this position [5]. On the other hand, the results obtained show that neither the implant height, nor the implant diameter (nor both parameters considered simultaneously) have an influence on the ISQ values when the RFA records are measured directly to the implants or using the transepithelial abutments [38,39,40].

The definition of early loading is not based on specific biological criteria, and the timing can make a significant difference in the stages of healing [41]. Therefore, the use of transepithelial abutments of different heights (on which the SmartPeg can be screwed), placed on the day of surgery and maintained throughout the healing process, allows the implants to be monitored during the descent and subsequent ascent of implant stability, applying a minimum torque (4–5 N) [25], instead of the 10N needed to unscrew a conventional transepithelial abutment to perform RFA directly to the implant platform, thus minimizing the risk of implant failure [40,41,42,43,44,45]. The implementation of an RFA measurement protocol through transepithelial abutments can decrease the pre-established loading times and therefore produce an improvement in the implant treatment quality [38]. Regarding the use of this prototype abutment in the different loading protocols, its use is indicated preferentially in the early loading, being able to measure RFA from 0 to 3 months after the placement of the implant without removing the healing abutment to check if optimal loading values have been obtained [17]. This individualized protocol can be carried out due to the fact that the results of the RFA records measured on transepithelial abutments of different heights (2, 3.5 and 5 mm) are comparable with those recorded directly to the implant.

However, it is necessary to point out that this transepithelial abutment prototype is designed exclusively for Klockner Essential Cone implants and validated for them. It is demonstrated that ISQ values are not influenced by increasing the EIL up to 1.8 mm; therefore, it could be applied to other implant systems as long as the abutment and SmartPeg design requirements are maintained, and the results obtained in this study suggest the possibility that this RFA record-taking work philosophy could be developed by other implant systems.

It should be said that the use of these new transepithelial abutments and their clinical application can be carried out when two-stage surgery or immediate implant restoration are not required. The promising results obtained in this study open new lines of research that could validate RFA measurements through transepithelial prosthetic abutments to monitor implant stability during early healing. This could allow to monitor implants subjected to immediate loading with provisional prosthesis as well as any implant loaded with definitive prosthesis restored with transepithelial prosthetic abutments without the need to unscrew them.

## 5. Conclusions

This in vivo study concludes that the RFA records obtained with Osstell ISQ through transepithelial abutments of different heights have a high reproducibility and repeatability. The implant height and diameter do not influence the reliability of the measurements. Therefore, measurements of implant stability obtained through prototype transepithelial abutments are highly reliable.

## Figures and Tables

**Table 1:** Mean implant stability quotients (ISQ) values and SD according to Osstell´s probe position achieved during the first and the repeated measurements *p* < 0.001 (CI 95%).

SmartPeg Screwed	Mesial		Distal		Buccal		Palatal/Lingual	
	First	Repeated	First	Repeated	First	Repeated	First	Repeated
Direct to implant	7.147 ± 5.887	73.095 ± 5.866	72.914 ± 6.071	72.724 ± 6.278	71.698 ± 6.804	71.655 ± 6.836	72.138 ± 6.589	72.30138 ± 6.414
To 2 mm abutment	73.56 ± 6.57	73.483 ± 7.94	73.595 ± 6.542	73.24 ± 6.298	71.69 ± 8.205	72.112 ± 7.922	72.164 ± 7.097	72.793 ± 6.914
To 3.5 mm abutment	73.345 ± 6.409	73.207 ± 6.169	73.19 ± 6.482	72.845 ± 6.389	71.259 ± 8.02	71.362 ± 7.909	72.112 ± 6.995	71.802 ± 6.935
To 5 mm abutment	72.483 ± 6.239	72.345 ± 6.347	71.983 ± 6.911	72.103 ± 6.771	69.828 ± 9.651	69.819 ± 9.32	71.578 ± 6.966	71.526 ± 7.012

**Table 2:** Intraclass Correlation Coefficients (ICC) according to Osstell´s probe orientation obtained during the first and the second measurement for each experimental group *p* < 0.001 (CI 95%).

	Mesial	Distal	Buccal	Palatal/Lingual
Direct to implant	0.993	0.985	0.980	0.970
	(0.989–0.995)	(0.978–0.990)	(0.971–0.986)	(0.957–0.979)
To 2 mm abutment	0.841	0.982	0.973	0.972
	(0.771–0.890)	(0.975–0.998)	(0.962–0.982)	(0.959–0.980)
To 3.5 mm abutment	0.990	0.98	0.966	0.981
	(0.985–0.993)	(0.972–0.986)	(0.951–0.977)	(0.972–0.987)
To 5 mm abutment	0.993	0.993	0.986	0.979
	(0.990–0.995)	(0.990–0.995)	(0.980–0.991)	(0.970–0.986)

**Table 3:** ICC value achieved according to implant length *p* < 0.001 (CI 95%).

	Mesial	Distal	Buccal	Palatal/Lingual
8 mm implant	0.99	0.996	0.993	0.921
	(0.995–1.00)	(0.980–0.999)	(0.968–0.998)	(0.648–0.982)
10 mm implant	0.992	0.979	0.968	0.965
	(0.986–0.995)	(0.996–0.987)	(0.948–0.980)	(0.943–0.979)
12 mm implant	0.993	0.993	0.996	0.985
	(0.988–0.996)	(0.986–0.996)	(0.993–0.998)	(0.972–0.992)

**Table 4:** ICC value achieved according to implant diameter *p* < 0.001 (CI 95%).

	Mesial	Distal	Buccal	Palatal/Lingual
3.5 mm	0.995	0.970	0.949	0.958
	(0.992–0.997)	(0.947–0.983)	(0.909–0.971)	(0.926–0.977)
4 mm	0.990	0.989	0.991	0.983
	(0.983–0.994)	(0.982–0.994)	(0.984–0.995)	(0.970–0.990)
4.5 mm	0.996	0.995	0.994	0.941
	(0.989–0.999)	(0.986–0.998)	(0.984–0.998)	(0.831–0.979)

**Table 5:** ICC value achieved according to Osstell probe orientation, in relation to the different abutment heights between them *p* < 0.001 (CI 95%).

	Mesial	Distal	Buccal	Palatal/lingual
Direct to implant versus 2-mm abutment	0.884	0.897	0.852	0.878
	(0.833–0.920)	(0.851–0.928)	(0.786–0.897)	(0.823–0.915)
Direct to implant vs. 3.5-mm abutment	0.924	0.921	0.845	0.906
	(0.891–0.948)	(0.885–0.945)	(0.776–0.892)	(0.865–0.935)
Direct to implant vs. 5-mm abutment	0.945	0.928	0.794	0.917
	(0.921–0.962)	(0.896–0.950)	(0.703–0.857)	(0.880–0.943)
2-mm versus 3.5-mm abutment	0.945	0.977	0.983	0.962
	(0.921–0.962)	(0.967–0.984)	(0.976–0.988)	(0.945–0.974)
2-mm versus 5-mm abutment	0.931	0.965	0.850	0.959
	(0.900–0.952)	(0.950–0.976)	(0.784–0.896)	(0.941–0.972)
3.5-mm versus 5-mm abutment	0.976	0.972	0.863	0.972
	(0.966–0.984)	(0.959–0.980)	(0.803–0.905)	(0.960–0.981)
